# Serial nitrogen-phosphate co-limitation controls the primary productivity in the transitional waters of northern South China Sea and the Pearl River Estuary

**DOI:** 10.3389/frmbi.2025.1655960

**Published:** 2025-10-08

**Authors:** Yuanhao Liu, Xunying Zhou, Ruoyu Niu, Rongman Yan, Shuaishuai Xu, Kangli Guo, Jing Guo, Jianchang Tao, Sha Wu, Shengwei Hou

**Affiliations:** Department of Ocean Science and Engineering, Southern University of Science and Technology, Shenzhen, China

**Keywords:** nitrogen limitation, Pearl River Estuary, South China Sea, primary productivity, cyanobacteria

## Abstract

Nitrogen (N) and phosphorus (P) are essential nutrients for marine phytoplankton, playing a crucial role in shaping the structure of microbial communities. Nutrients in coastal seawater are influenced by multiple factors, including ocean currents, terrestrial runoff, and anthropogenic activities, leading to region-specific patterns of nutrient limitation. This study investigates nutrient limitation in the transitional waters near Sanmen Island, located at the confluence of the Pearl River Estuary (PRE) and the northern South China Sea. Using 4-hourly *in situ* time-series observations and nutrient addition experiments, we found that nitrogen limitation persists in this region despite its proximity to the nutrient-rich Pearl River. Urea addition significantly enhanced primary productivity, as evidenced by the increased chlorophyll *a* concentration and the increased relative abundance of cyanobacteria, whereas phosphate addition alone favored the growth of heterotrophic bacteria, yet limited the growth of cyanobacteria and other primary producers. Combined nitrogen-phosphorus treatments revealed serial co-limitation, where nitrogen relief shifted limitation to phosphorus. In conclusion, these findings highlight the complex nutrient dynamics in transitional coastal waters and underscore the impact of anthropogenic nutrient discharge on ecosystem productivity.

## 1 Introduction

Marine phytoplankton are fundamental to oceanic food webs and global biogeochemical cycles, contributing approximately 50% of global primary productivity (Falkowski et al., 1998; Field et al., 1998). The availability of key inorganic nutrients primarily governs the growth and community structure of marine phytoplankton, and their cellular stoichiometry roughly follows the Redfield ratio, approximating the average ratio of inorganic C, N, and P found in the ocean (Redfield, 1958). Coastal marine environments exhibit complex and spatially variable patterns of nutrient limitation that differ significantly from those in open ocean systems (Elser et al., 2007). While oligotrophic open ocean regions typically experience N limitation or N-P co-limitation, coastal waters present a more heterogeneous picture due to the confluence of terrestrial inputs, anthropogenic activities, and oceanographic processes, leading to a notable deviation in inorganic N:P ratios from the Redfield ratio (Peng et al., 2002; Elser et al., 2007; Lan et al., 2024). According to Liebig’s Law of the Minimum, the primary productivity of marine phytoplankton is limited by the most scarce essential nutrient in the environment (de Baar, 1994). Nitrogen is often the primary limiting nutrient in marine environments, a pattern consistently observed across diverse coastal systems (Vitousek and Howarth, 1991). However, modern understanding recognizes that multiple essential nutrients can be deficient in the system, resulting in simultaneous co-limitation (where multiple nutrients are equally limiting at the same time) or serial co-limitation (where the relief of one limiting nutrient shifts the limitation to another) (de Baar, 1994; Harpole et al., 2011; Moore et al., 2013). A global analysis of ocean phytoplankton nutrient limitation has revealed a high prevalence of co-limitation in coastal regions, where N, P and other nutrients can simultaneously or sequentially limit primary productivity (Elser et al., 2007; Moore et al., 2013; Cao et al., 2024). This complexity is particularly pronounced in transitional coastal waters where multiple water masses converge, creating dynamic conditions that can rapidly alter nutrient availability and limitation patterns (Nixon, 1995), particularly in the face of unprecedented pressures from climate change, eutrophication, and human activities.

The northern South China Sea (SCS) is one of the most dynamic subtropical coastal marine systems globally, characterized by complex interactions between monsoon-driven circulation, river inputs, and anthropogenic activities (Liu et al., 2002). Daya Bay is located in the northern SCS off the coast of Shenzhen and Huizhou. Within the bay, submarine groundwater discharge contributes more nutrients than riverine inputs (Wang et al., 2018). Nitrate and nitrite primarily originate from atmospheric deposition and submarine groundwater discharge, while dissolved inorganic phosphorus (DIP) is mainly derived from riverine sources, and silicate is predominantly supplied by groundwater discharge from the seabed and continental slope (Wang et al., 2018). The rapid economic development along the coast of Shenzhen and Huizhou, such as tourism, fishery, aquaculture, has led to an increasingly anthropogenic impact on Daya Bay and nearby coastal waters, including eutrophication, an increase in the N:P ratio, a shift in the limiting nutrient factor from N to P (Wang et al., 2004, 2008; Wu et al., 2009; Shi and Huang, 2013; Guo et al., 2023). Eutrophication leads to an increase in primary productivity, which has had a significantly negative impact on the aquaculture and tourism industries of Daya Bay (Wu et al., 2009).

Sanmen Island is situated at the mouth of Daya Bay in northern SCS, near the Pearl River Estuary (PRE), making it a unique transitional zone where multiple water masses converge. Seawater environmental parameters near Sanmen Island are influenced by multiple factors, including tidal dynamics from SCS, groundwater discharge from the continent and island, riverine input and human activities (Wang et al., 2018; Li et al., 2021). During the wet season, typically from April to September, the increased flow of the Pearl River can introduce substantial nutrient loads into the nearby estuarine and coastal waters (Yin and Harrison, 2008). The summer riverine input typically enhances nitrogen concentration in the estuary, though phosphorus concentration remains low, and the interaction with seawater from the northern South China Sea further complicates the nutrient limitation in this highly dynamic system (Li et al., 2017). Due to their different geographical locations, the PRE and Daya Bay exhibit distinct patterns of nutrient limitation in summer. The N:P ratio of surface water in the PRE is significantly influenced by Pearl River input and coastal anthropogenic activities (Niu et al., 2020; Tao et al., 2021), while Daya Bay is primarily influenced by Guangdong coastal upwelling brought by the southwest monsoon, with relatively weaker impact from terrestrial runoff and human influence (Han and Ma, 1988; Li et al., 1990; Zhang, 1992; Yang and Tan, 2019). While in the transitional zone offshore Sanmen Island, in the vicinity of the Pearl River Estuary and Daya Bay, the bioavailability of nutrients depends on the mixing of different water bodies, creating a natural laboratory for studying nutrient limitation patterns. The frequent occurrence of phytoplankton blooms in this region during spring and summer is consistent with seasonal variations in nutrient availability and productivity (Lin et al., 2024). However, the effect of nutrient limitation on phytoplankton growth, particularly regarding the role of organic nitrogen sources and the competitive dynamics between autotrophs and heterotrophs, remains poorly understood. Altogether, Sanmen Island’s unique geographical location and complex hydrological conditions make it a special site for studying the ecology of transitional waters.

This study aims to elucidate the primary nutrient limitation in these transitional waters, focusing on the role of organic nitrogen sources like urea on phytoplankton growth, as well as the interactive relationship between nutrient limitation and microbial community structure. Specifically, in July 2022, an *in situ* observation and an incubation experiment with urea and potassium dihydrogen phosphate (KH_2_PO_4_) additions at Sanmen Island were conducted. Through 16S rRNA gene and metagenomic sequencing, the following questions will be addressed: (1) What is the nutrient limitation pattern in the Sanmen Island region? (2) How do microbial communities respond to the addition of urea and KH_2_PO_4_, respectively?

## 2 Materials and methods

### 2.1 Experimental design

**Figure 1:**
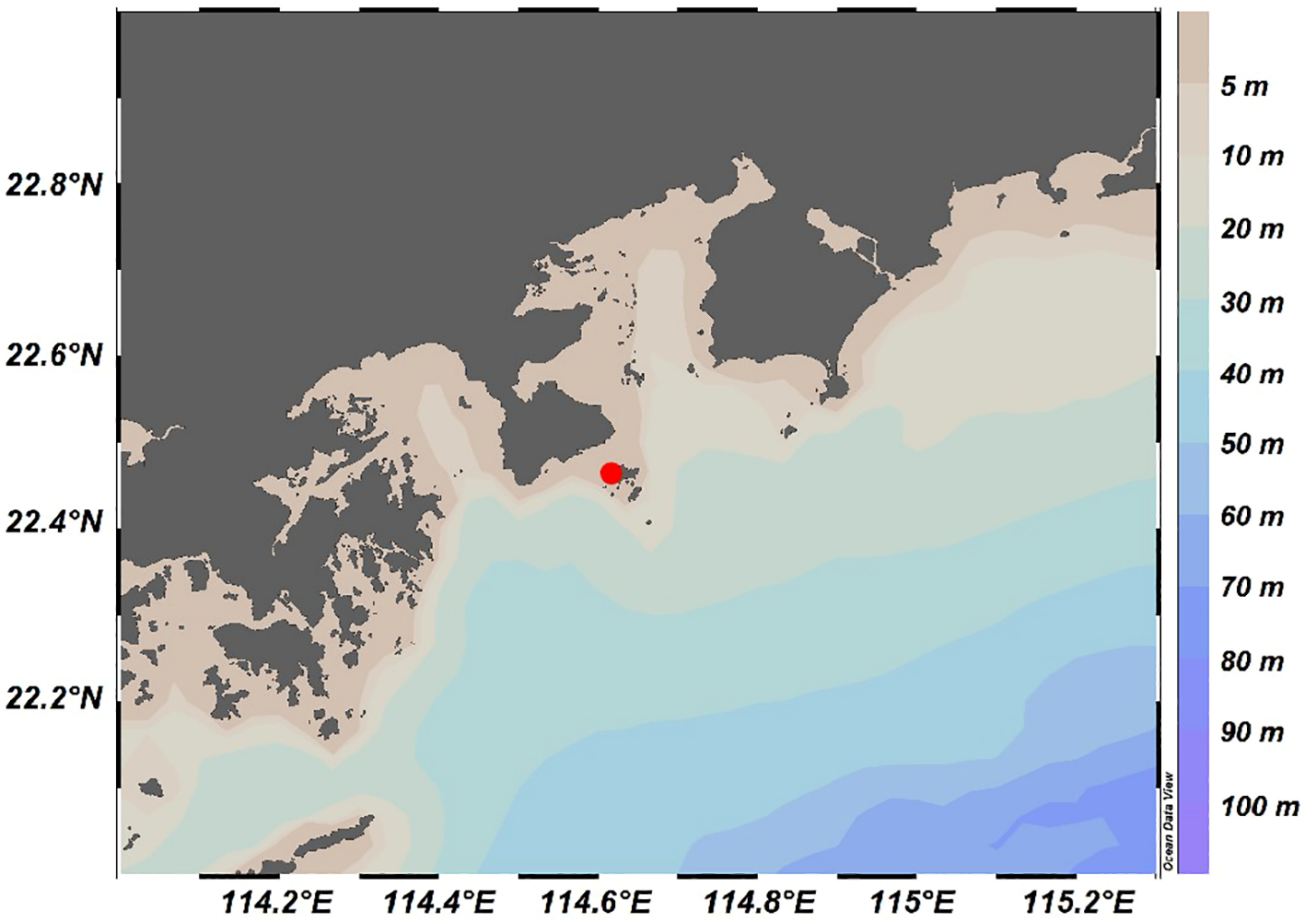
The sampling location (red dot) off the Sanmen Island, in the south of Daya Bay.

The sampling site was located at Mawan Wharf, Sanmen Island, with coordinates of 22°27′49.65″N, 114°37′0.73″E (**Figure 1**). This location was specifically chosen as a representation of transitional coastal waters, as it experiences the confluence of multiple water masses with more pronounced nutrient dynamics compared to inner bay or riverine locations. The experimental design consisted of two complementary approaches, a high-frequent *in situ* observation and a controlled nutrient incubation experiment. For the *in situ* observation, samples were collected from surface seawater (~20 cm depth) between 22:00 on July 18, 2022, and 14:00 on July 21, 2022, with the sampling intervals of 4 hours, yielding a total of 17 sample sets. Detailed sampling time for each sample is provided in **Supplementary Table 2**. Meanwhile, an incubation experiment was initiated at 18:00 on July 18, 2022. Considering that Sanmen Island is directly influenced by anthropogenic activities, and referring to previous incubation experiments, we selected organic nitrogen compound urea and inorganic phosphate KH_2_PO_4_ as the added nitrogen and phosphorus sources, respectively (Rondell et al., 2000; Bonnet et al., 2016). Initial surface seawater from the observation site was transferred into four sets of three replicate 20 L transparent low-density polyethylene buckets. The four groups were: (1) Blank group, the control group without nutrient addition; (2) N group, with urea added to reach a final concentration of 0.16 mmol urea-N/L; (3) P group, with KH_2_PO_4_ added to a final concentration of 0.01 mmol KH_2_PO_4_-P/L; (4) NP group, with both urea and KH_2_PO_4_ added at the same concentrations as the N and P groups. Here, the final concentration of phosphate was moderately higher than the highest observed value in surface seawater of Daya Bay during summer (Li et al., 2019). The urea concentration was determined based on the final concentration of phosphate and the Redfield ratio (Redfield, 1958). Due to tidal influence, one replicate each from the Blank and N groups was damaged during the *in situ* incubation. The remaining samples were collected after 68 hours for productivity and microbial composition analysis. Before sample collection, *in situ* measurements of temperature, pH, dissolved oxygen (DO), salinity, and chlorophyll *a* concentration were taken. For microbial sample collection, large particles and zooplankton were first removed by pre-filtering through a 200-mesh (74 μm) nylon sieve. Then, approximately 20 liters of seawater were filtered through 0.22 μm pore size polycarbonate (PC) membranes (Millipore, USA) to capture prokaryotic cells. The filters were used to extract DNA. The remaining filtrate was analyzed to measure the concentrations of nutrients (including nitrate, nitrite, ammonia, phosphate, and silicate) and dissolved organic carbon (DOC).

### 2.2 Measurement and analysis of environmental parameters

Temperature, pH, DO, salinity, and chlorophyll *a* concentration were measured *in situ* using a MultiAnna MTA-6A multi-parameter water quality sonde (Lightsun, China). During measurement, the sonde was immersed in seawater and continuously monitored for at least 3 minutes. After removing outliers, the arithmetic mean of the remaining data was recorded as the result of the environmental parameter.

DOC concentration was measured with a TOC-L total organic carbon analyzer (Shimadzu, Japan) according to the manufacturer’s protocol. Nutrient concentrations, including nitrate, nitrite, ammonia, silicate, and phosphate, were measured using the CleverChem Anna automatic discrete analyzer (DeChem-Tech, Germany) according to the “GB17378.4, 2007 Specifications for oceanographic survey-part 4: Survey of chemical parameters in sea water” and instrument operation manual (GB17378.4, 2007). Data analysis and visualization were conducted using R packages with R version 4.2.3 (R Core Team, 2023). Specifically, *in situ* samples grouping was determined using the mclust package based on the temporal variation of nutrient concentrations (Scrucca et al., 2016). The Wilcoxon test was used to verify the validity of samples grouping (Wilcoxon, 1945). The ggplot2 package (version 3.5.0) was used for data visualization (Wickham, 2016).

### 2.3 DNA extraction and sequencing

FastDNA™ SPIN Kit for Soil (MP Biomedical, USA) was used to extract microbial DNA from filter membranes. DNA samples were sent to Novogene Co., Ltd. (Tianjin, China) for amplicon and metagenomic sequencing. The V4-V5 region of prokaryotic 16S rRNA gene was amplified using primers 515Y (5’-GTGYCAGCMGCCGCGGTAA-3’) and 926R (5’-CCGYCAATTYMTTTRAGTTT-3’) (Yeh et al., 2021). The PCR amplification steps include: (1) pre-denaturation at 95°C for 120 seconds; (2) 30 cycles of denaturation at 95°C for 45 seconds, annealing at 50°C for 45 seconds and extension at 68°C for 90 seconds; (3) final extension at 68°C for 300 seconds (Yeh et al., 2021). Amplicon sequencing was carried out on the Illumina NovaSeq PE250 platform (Illumina, USA). Metagenomic sequencing was performed on the Illumina NovaSeq PE150 platform (Illumina, USA).

### 2.4 16S rRNA gene sequencing analysis

The amplicon sequencing data were processed on the QIIME2 platform (Bolyen et al., 2019). Adapter and primer sequences in the raw reads were trimmed using Cutadapt (version 4.6) (Martin, 2011). The trimmed sequences were classified into prokaryotic and eukaryotic sequences using BBSplit (BBTools version 39.19, https://sourceforge.net/projects/bbmap/) with reference to the SILVA 132 and PR2 4.14.0 databases (Quast et al., 2012; Guillou et al., 2012). For prokaryotic sequences, denoising, paired-end reads merging, and clustering were performed using DADA2 (version 1.22.0) to obtain amplicon sequence variants (ASVs) (Katoh et al., 2002; Callahan et al., 2016). Taxonomic annotation of ASVs was conducted according to the SILVA 138.1 database (Quast et al., 2012). ASVs annotated as chloroplasts or mitochondria were discarded. Default parameters were used for all the software unless otherwise specified. The quality control results are detailed in **Supplementary Table 1**. Statistical analysis was performed in QIIME2 and R software (R version 4.2.3) (Bolyen et al., 2019; R Core Team, 2023). All samples were rarefied to 140,168 sequences per sample to reduce the impact of varying sequencing depths (**Supplementary Figure 1**). Alpha diversity of prokaryotes was assessed using the Simpson index. A significant test of alpha diversity was conducted via the Wilcoxon test (Wilcoxon, 1945). Beta diversity was evaluated using non-metric multidimensional scaling (NMDS) analysis via the vegan package (version 2.6-4), and the significance test between different groups was conducted using analysis of similarity (ANOSIM) via the phyloseq package (version 1.42.0) (Clarke and Green, 1988; McMurdie and Holmes, 2013; Oksanen et al., 2022). Differential abundance analysis (DAA) was performed using ANCOM-BC (version 1.0) (Lin and Peddada, 2020). Centered log-ratio (CLR) transformation and Pearson correlation were used to analyze the correlation between cyanobacteria and chlorophyll *a* concentration (Aitchison, 1982; Rodgers and Nicewander, 1988). Data visualization was conducted using ggplot2 (version 3.5.0) (Wickham, 2016). When plotting the composition of the prokaryotic community, taxa with an average relative abundance <1% across groups were grouped into “Others”. This approach was also used in DAA and correlation analysis between cyanobacteria and chlorophyll *a* concentration to minimize interference from low-abundance taxa. Given the marine environment, all taxa annotated as freshwater species were labeled with the “-like” suffix to suggest close relatives of marine environments.

### 2.5 Metagenomic analysis

For raw sequencing data, Readfq V8 (https://github.com/lh3/readfq) and Bowtie2 (version 2.2.4) were used for quality control and host contamination removal, respectively (Langmead and Salzberg, 2012). Clean reads were assembled into contigs using MEGAHIT (version 1.2.9; parameters: –min-count 2 –k-list 21,33,55,77,99,127 –min-contig-len 1000) (Li et al., 2015, 2016). To obtain more functional genes and more precise taxonomy annotation of functional genes, single-sample binning and co-binning were performed with BASALT (version 1.0.0; parameters: –max-ctn 10 –min-cpn 50 –mode continue) to obtain metagenome-assembled genomes (MAGs) (Qiu et al., 2024). Specifically, co-binning was conducted based on the clustering result of sourmash (version 4.8.8) (Brown and Irber, 2016). Samples clustered into the same group were co-binned together to recover more MAGs. MAGs were dereplicated at an average nucleotide identity (ANI) of 95% using dRep (version 3.4.5) (Olm et al., 2017). The completeness and contamination of MAGs were assessed again using CheckM (version 1.2.2) (Parks et al., 2015). 202 MAGs with completeness > 50% and contamination < 10% were retained for downstream analysis. The data preprocessing results are detailed in **Supplementary Table 1**. Taxonomic annotation of MAGs was conducted using GTDB-Tk (version 2.3.2), and the result was translated into NCBI taxonomy classification via gtdb_to_ncbi_majority_vote.py (v0.2.1, https://github.com/Ecogenomics/GTDBTk) (Chaumeil et al., 2019, 2022). Taxonomic annotation of contigs was conducted via MEGAN6 (version 6.24.20) against the NCBI nt database (downloaded on June 10, 2022) and megan-nucl-Feb2022.db (https://software-ab.cs.uni-tuebingen.de/download/megan6/) (Huson et al., 2016; Sayers et al., 2022). Coding DNA Sequences (CDS) of contigs and MAGs were predicted using Prokka (version 1.14.6), followed by functional annotation against the KEGG database (version 106.0) using KofamScan (version 1.3.0) (Seemann, 2014; Aramaki et al., 2019). CDS and predicted protein sequences were clustered at 95% sequence identity using CD-HIT (version 4.8.1) (Li and Godzik, 2006; Fu et al., 2012). To evaluate the microbial demand for nitrogen and phosphorus in different experimental groups, relative gene abundance related to the uptake and utilization of environmental nitrogen and phosphorus was analyzed. Transcript per million (TPM) was calculated using CoverM (version 0.7.0) with parameters: contig –mapper bwa-mem –min-read-percent-identity 0.95 –min-read-aligned-percent 0.75 -m tpm (Li and Dewey, 2011; Wagner et al., 2012; Li, 2013; Aroney et al., 2025). For phylogenetic tree construction, multiple sequence alignment (MSA) was first constructed using MUSCLE (version 5.1), followed by trimming of MSA using trimAl (version 1.4) with “-automated1” parameter (Capella-Gutiérrez et al., 2009; Edgar, 2022). Finally, the phylogenetic tree was constructed using IQ-TREE (version 2.2.0.3) with the parameter: -m MF (Nguyen et al., 2015). The base R (R version 4.2.3) and ggplot2 (version 3.5.0) packages were used for data visualization (Wickham, 2016; R Core Team, 2023).

## 3 Results

### 3.1 Nitrogen limitation controls the primary productivity in seawater near Sanmen Island

**Figure 2:**
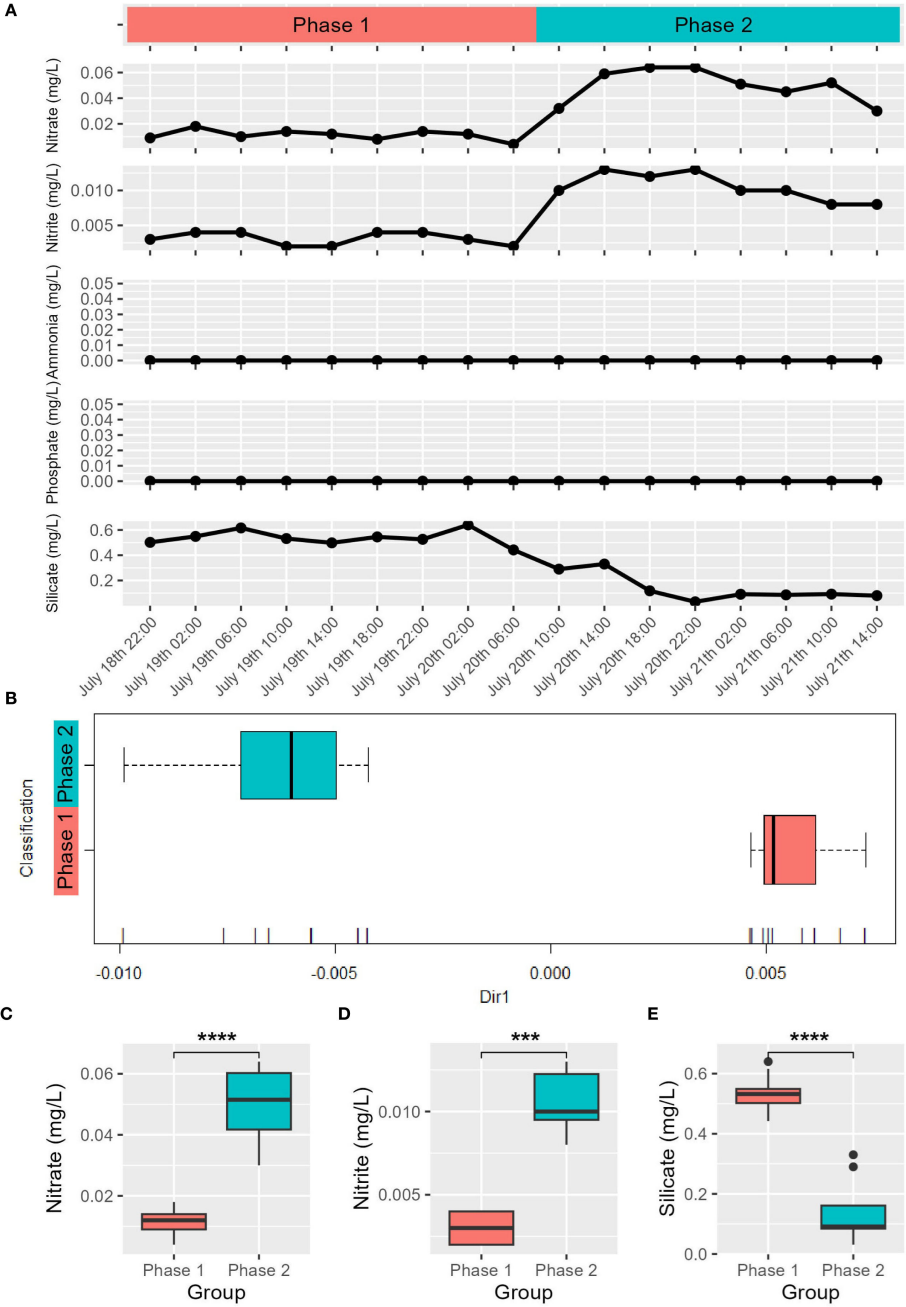
Variations in nutrient concentrations in the *in situ* environment **(A)** Grouping result of the *in situ* samples based on nitrate, nitrite and silicate concentrations **(B)** The horizontal axis represents the coordinate projection of data on a specific dimension (Dir1). The 17 vertical lines at the bottom of the B panel represent the projection of 17 *in situ* samples on Dir1, ordered left to right as: L2022, L2018, L2106, L2102, L2114, L2014, L2110, L2010, L1922, L1918, L2006, L1822, L1902, L1906, L1914, L1910, L2002; Wilcoxon test results of nitrate **(C)**, nitrite **(D)** and silicate **(E)** concentrations between Phase 1 and Phase 2. “***”*p*-value < 0.001, “****”*p*-value < 0.0001.

Phased variations were observed in nitrate, nitrite, and silicate concentrations in the *in situ* environment. Nitrate and nitrite concentrations remained relatively low before 06:00 on July 20 but increased significantly after 10:00 (*p*-value < 0.001), while the silicate concentration exhibited the opposite trend (**Figure 2A**). After sampling clustering using nitrate, nitrite, and silicate concentrations (see Methods), nine samples collected from 22:00 July 18 to 06:00 July 20 (L1822, L1902, L1906, L1910, L1914, L1918, L1922, L2002, L2006) were grouped as Phase 1, while eight samples from 10:00 July 20 to 14:00 July 21 (L2010, L2014, L2018, L2022, 2102, L2106, L2110, L2114) were grouped as Phase 2 (**Figure 2B**). Significant differences (*p*-value < 0.01) were observed between Phase 1 and Phase 2 for nitrite, nitrate, and silicate concentrations, strengthening the rationality of grouping (**Figures 2C–E**). The concurrent increases in pH, dissolved oxygen, and chlorophyll *a* are consistent with enhanced photosynthetic activity and primary productivity during Phase 2 (Hamdhani, 2024). The silicate depletion in Phase 2 may indicate that diatoms made a significant contribution to primary productivity. Phosphate and ammonia concentrations were below the detection limit of the instrument (**Figure 2A**). In the incubation experiment, urea addition alone or combined with KH_2_PO_4_ enhanced primary productivity. Chlorophyll *a* concentration in the Blank group ranged from 2.1997 to 3.1007 μg/L, while in the N group, they rose to between 5.3352 and 12.1482 μg/L, demonstrating that urea addition enhanced primary productivity. In the P group, chlorophyll *a* concentration remained almost unchanged compared to the Blank group, with a slight decrease in average value (**Supplementary Table 2**). The NP group yielded substantially higher chlorophyll *a* concentrations (16.0793-21.2664 μg/L), suggesting NP co-addition further improved the primary productivity than N addition alone. These results indicate that coastal surface seawater near Sanmen Island exhibited serial nitrogen-phosphate co-limitation, dominated by nitrogen limitation.

### 3.2 Distinct microbial diversity and composition patterns between groups

**Figure 3:**
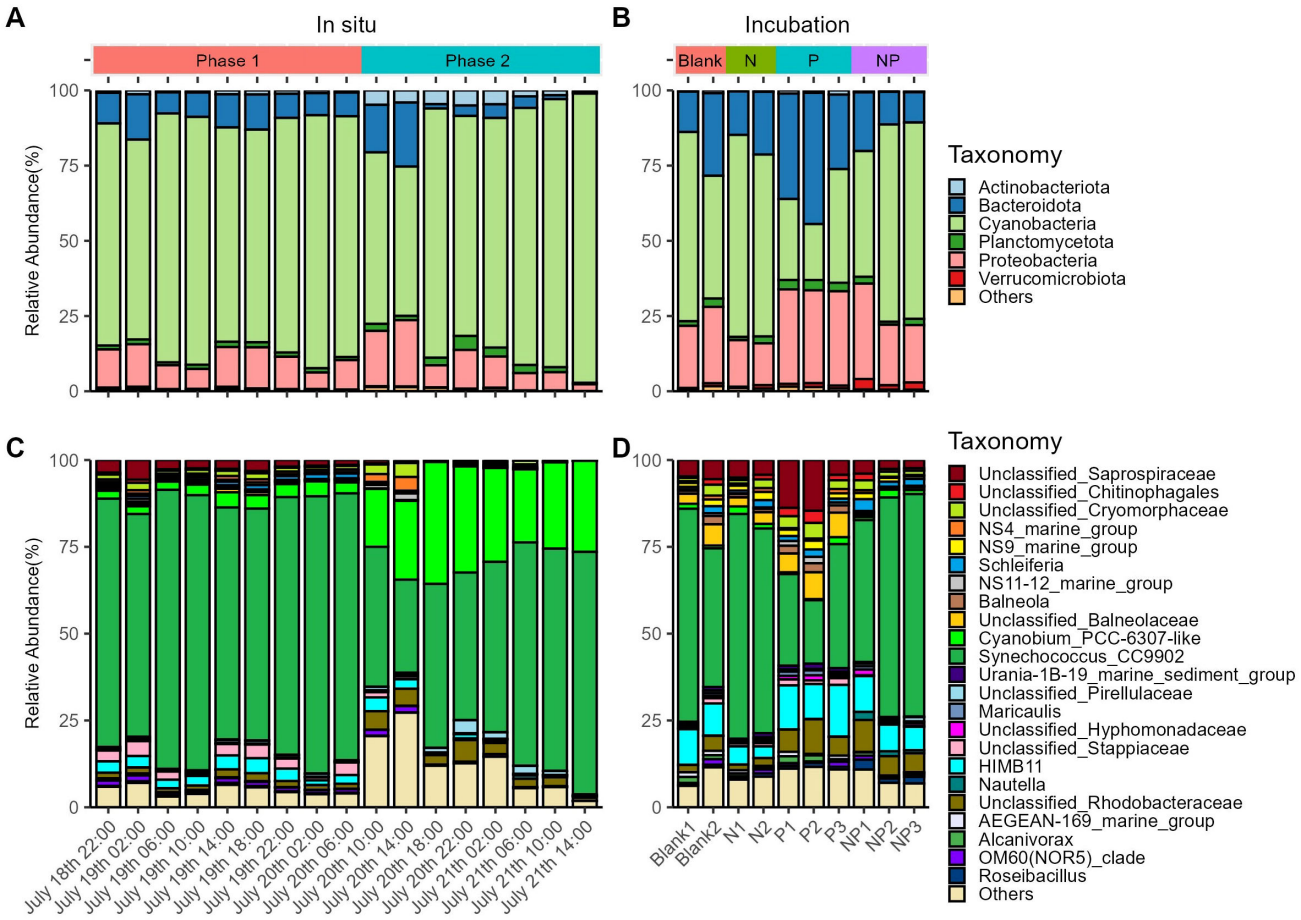
Prokaryotic community structure of the *in situ* environment **(A, C)** and the incubation experiment **(B, D)** at the phylum level **(A, B)** and other taxonomic levels **(C, D)**.

At the phylum level, Bacteroidota, Cyanobacteria, and Proteobacteria were the most abundant prokaryotes (**Figures 3A, B**). *In situ* observations showed that Cyanobacteria consistently dominated the prokaryotic community, while Actinobacteriota showed a significant increase in relative abundance in Phase 2 (*q*-value < 0.001) (**Figure 3A**; **Supplementary Table 3**). At the genus level, *Synechococcus*_CC9902 had the highest relative abundance, whereas *Cyanobium*_PCC-6307-like significantly increased in Phase 2 (*q*-value < 0.001), suggesting an ecotype transition between Phase 1 and Phase 2. Low-abundance taxa (Others) initially increased in Phase 2 but rapidly declined thereafter (**Figure 3C**). In the incubation experiment, Cyanobacteria did not always dominate the prokaryotic community. In the P group, the mean relative abundance of Bacteroidota and Proteobacteria both exceeded that of Cyanobacteria (**Figure 3B**). Additionally, Unclassified Saprospiraceae displayed higher mean abundance in the P group compared to the other three groups (**Figure 3D**). Unlike in the *in situ* environment, *Cyanobium_*PCC-6307-like remained at low relative abundance (<1.5%) across all groups (**Figure 3D**).

**Figure 4:**
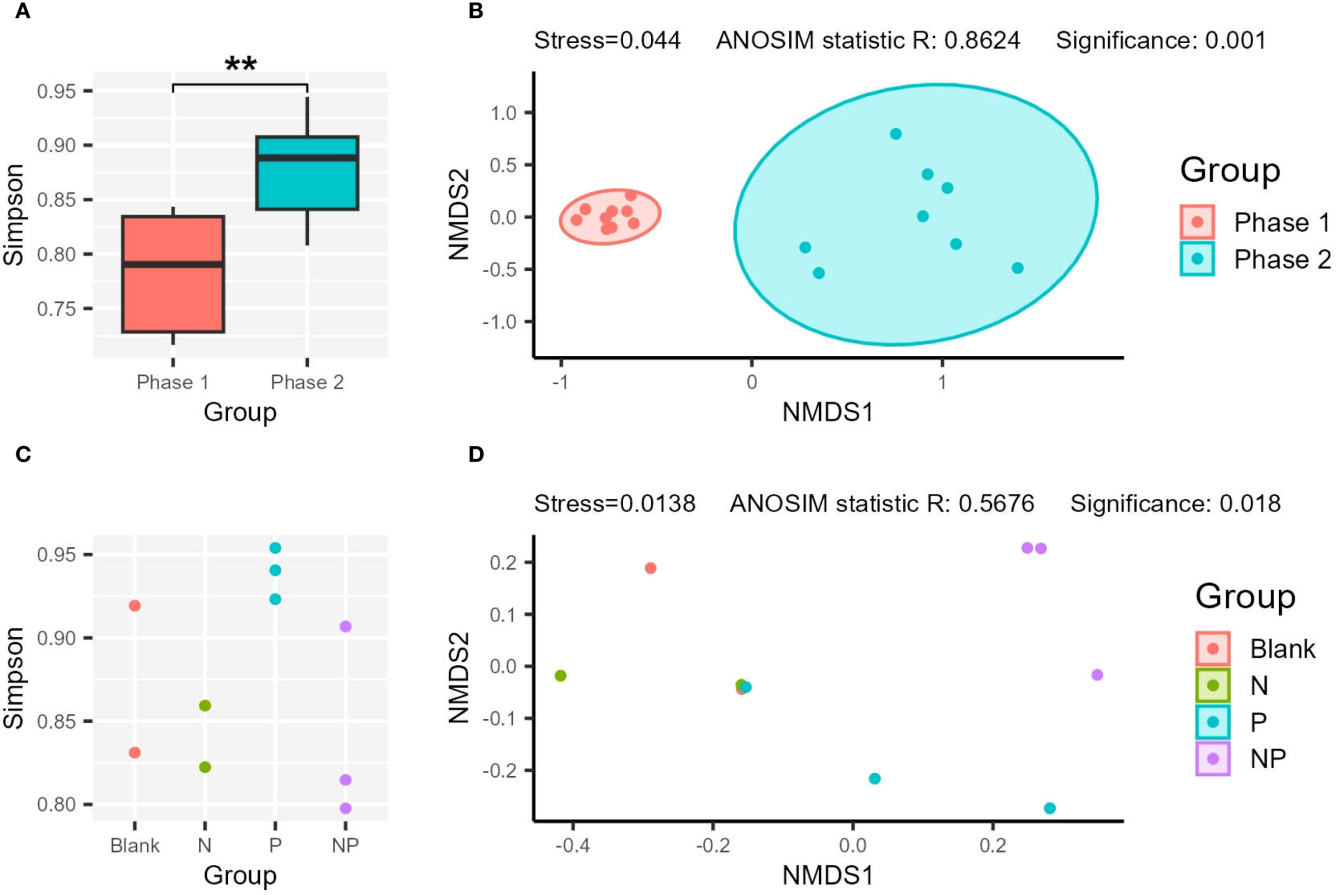
Comparison of the alpha and beta diversity. The comparison of Simpson index **(A, C)**, and NMDS analysis **(B, D)** of the *in situ* **(A, B)** and incubation samples **(C, D)**. “**”: p-value < 0.01.

For *in situ* samples, a significant difference in prokaryotic diversity was observed between Phase 1 and Phase 2. The diversity (Simpson index) of prokaryotes was significantly higher in Phase 2 compared to Phase 1 (*p*-value < 0.05) (**Figure 4A**). NMDS analysis also confirmed distinct clustering between Phase 1 and Phase 2 (Significance < 0.05) (**Figure 4B**). In the incubation experiment, the Blank, N, and NP groups exhibited larger fluctuations in diversity index, whereas the P group maintained relatively stable values and had the highest mean Simpson index (**Figure 4C**).

### 3.3 Contrasting microbial responses to urea and phosphate addition

**Figure 5:**
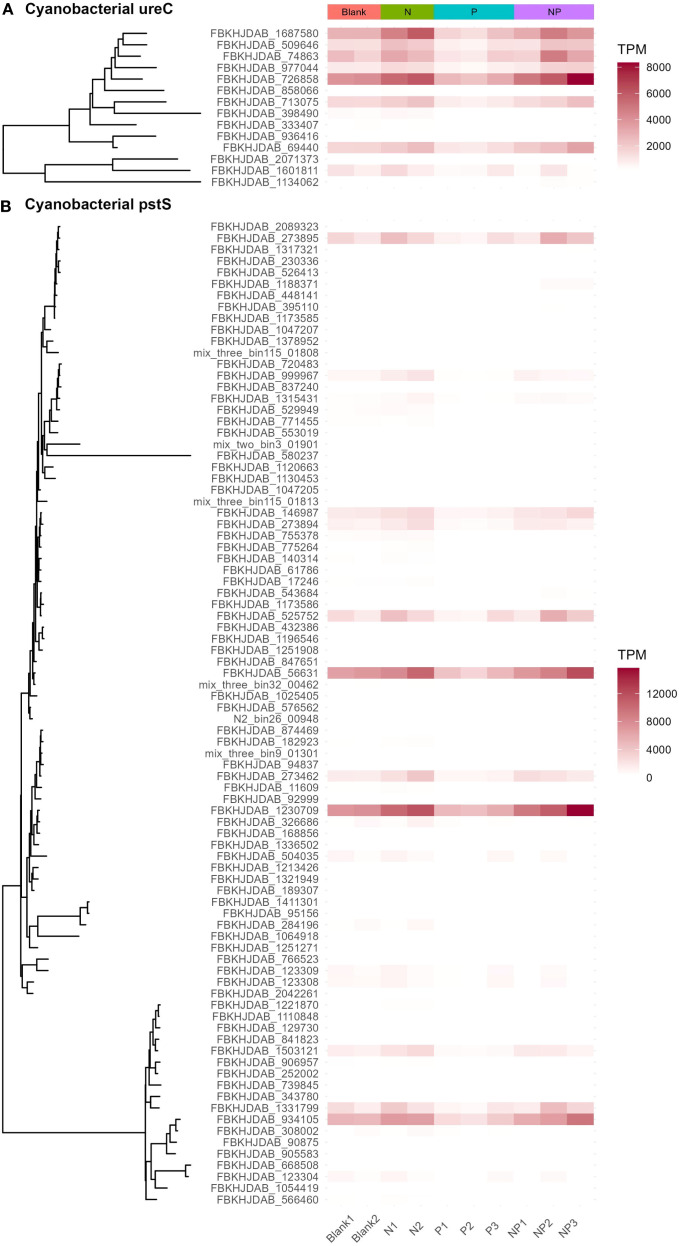
Taxonomic annotations and relative gene abundances of cyanobacterial *ureC* **(A)** and *pstS* **(B)**. Urease subunit alpha (*ureC*) and phosphate transport system substrate-binding protein (*pstS*) are marker genes for urea hydrolysis and phosphate transmembrane transport, respectively (Hung et al., 2013; Abdo et al., 2022).

The addition of urea promoted the utilization potential of nutrients by autotrophic microorganisms. In both the N and NP groups, the relative gene abundances of ferredoxin-nitrite reductase (*nirA*), ferredoxin-nitrate reductase (*narB*), alkaline phosphatase D (*phoD*), urease (*ureABCDEFG*), phosphate transport system (*pstSCAB*), phosphonate transport system (*phnC*, *phnD*, *phnE*), nitrate/nitrite transporters (*NRT2*, *narK*, *nrtP*, *nasA*), phosphate starvation-inducible proteins (*phoH*, *phoL*), urea transport system (*urtABCDE*), phosphonate dehydrogenase (*ptxD*), Trk/Ktr system potassium uptake proteins (*trkA, trkG, trkH, ktrA, ktrB, ktrC, ktrD*), and potassium/hydrogen antiporter (*cvrA, nhaP2*) derived from cyanobacteria increased. In contrast, this phenomenon was not observed in the P group. Instead, the abundances of the above-mentioned genes derived from cyanobacteria decreased slightly in the P group, indicating that the competitiveness of cyanobacteria in the P group decreased (**Figure 5** and **Supplementary Table 4**). Urea addition stimulated cyanobacteria to enhance their transport and utilization potential of dissolved inorganic nitrogen (DIN), urea, DIP, and phosphonate, thereby increasing primary productivity. The phosphate transport system substrate-binding protein (*pstS*), a key component of the phosphate transport system, was found to be upregulated under phosphorus starvation (Willsky and Malamy, 1980; Ames, 1986; Hussein et al., 2020). In the N group, the relative gene abundance of cyanobacterial *pstS* increased, indicating a higher potential demand for phosphorus by cyanobacteria. This result also supports the notion that the nutrient limitation pattern in the seawater environment of Sanmen Island is dominated by the serial nitrogen limitation.

The addition of phosphate enhanced the potential of heterotrophic bacteria to compete for nitrogen and phosphorus. Chlorophyll *a* concentration did not increase in the P group, nor did the abundances of cyanobacterial genes relate to nitrogen and phosphorus transport and utilization. However, in the P group, heterotrophic bacteria exhibited an enhanced potential for nutrient competition under phosphate stimulation. Compared with the Blank group, the relative gene abundances of alkaline phosphatases (*phoA*, *phoB*), alkaline phosphatase D (*phoD*), urease (*ureABCDEFG*), phosphate transport system (*pstSCAB*), phosphate transport system protein (*phoU*), phosphonate transport system (*phnC*, *phnD*, *phnE*, *phnF*, *phnK*), phosphate-Na^+^ symporter (*yjbB*), phosphate starvation-inducible proteins (*phoH*, *phoL*), phosphate regulon sensor histidine kinase (*phoR*), phosphate regulon response regulator (*phoB*), nitrate/nitrite transport system (*nrtA*, *nasF*, *cynA*, *nrtB*, *nasE*, *cynB*), phosphonate dehydrogenase (*ptxD*), Trk/Ktr system potassium uptake proteins (*trkA, trkG, trkH, ktrA, ktrB, ktrC, ktrD*), KUP system potassium uptake protein (*kup*), potassium-dependent mechanosensitive channel (*mscK, kefA, aefA*), inward rectifier potassium channel (IRPC), voltage-gated potassium channel (*kch, trkA, mthK, pch*), and glutathione-regulated potassium-efflux system ancillary protein KefG (*kefG*) derived from heterotrophic bacteria, mainly Rhodobacterales and Maricaulales, increased in the P group. Among these genes, nitrate/nitrite transport system related genes and some *phoD* genes also increased in the NP group, while other genes remained at the same level or decreased (**Supplementary Table 4**). In contrast, the abundances of the above genes derived from heterotrophic bacteria decreased in the N group, indicating that the heterotrophic competition for nutrients was weaker in the N group.

## 4 Discussion

Studies on marine eutrophication confirm that increases in nitrogen levels can significantly boost algal growth, potentially triggering harmful algal blooms (Lan et al., 2024). Urea has gained recognition as a significant nitrogen source for marine phytoplankton, including the picocyanobacteria *Synechococcus* (Solomon et al., 2010). The ability of marine microorganisms to utilize urea through urease enzymes provides them with a competitive advantage in nitrogen-limited environments, contributing to their ecological success in coastal waters (Dyhrman and Anderson, 2003; Mulholland and Lee, 2009). While nitrogen is often the primary limiting nutrient in marine systems, phosphorus plays a critical role in freshwater and can significantly influence marine ecosystems, particularly in coastal lagoons and estuaries with elevated phosphorus inputs from agriculture and sewage (Sylvan et al., 2006; Elser et al., 2007; Conley et al., 2009). Phosphorus limitation is less common in marine systems but becomes more prominent when nitrogen levels are already elevated, making freshwater-influenced marine systems vulnerable to phosphorus-driven changes in community structure (Sylvan et al., 2006; Elser et al., 2007). Heterotrophic bacteria exhibit higher phosphorus demand compared to autotrophic organisms, with their phosphate uptake accounting for a substantial portion of total phosphate uptake in marine environments (Cotner and Wetzel, 1992). This competitive advantage allows heterotrophic bacteria to thrive when phosphate is added, potentially limiting the growth of primary producers like cyanobacteria. These dynamics highlight the significance of understanding phosphorus cycling and competition in influencing microbial community structure and nutrient availability in coastal ecosystems.

Daya Bay exhibits distinct nutrient limitation factors between the inner and outer bay. Prior to China’s Reform and Opening-Up, the inner bay was less impacted by human activities and showed nitrogen limitation (Wang et al., 2004, 2008; Shi and Huang, 2013). With subsequent coastal economic development and increased anthropogenic influences, the limiting nutrient factor in the inner bay shifted from nitrogen to phosphorus (Wang et al., 2004, 2008; Shi and Huang, 2013; Guo et al., 2023). However, in areas less affected by human activities, such as the bay mouth, nitrogen remains the limiting element factor (Ma et al., 2023; Zhao et al., 2025). The Pearl River Estuary and Daya Bay, located west and east of Shenzhen, respectively, demonstrate different patterns of nutrient limitation. In summer, the N/P ratio of surface water in the PRE exhibits a “high at north and south, low in the middle” pattern, which is significantly influenced by the Pearl River input and coastal anthropogenic activities (Li et al., 2017; Ke et al., 2022). Although Daya Bay also receives terrestrial runoff and human influence, their impacts are relatively weaker and diluted by Guangdong coastal upwelling (Han and Ma, 1988; Li et al., 1990; Zhang, 1992; Yang and Tan, 2019). During summer, influenced by the southwest monsoon, Guangdong coastal upwelling invades Daya Bay (Han and Ma, 1988; Li et al., 1990; Han and Ma, 1991; Zhang, 1992; Xu et al., 2014; Yang and Tan, 2019). Given the relatively small discharge from Daya Bay’s coastal rivers, the intensity of Guangdong coastal upwelling primarily determines the spatial distribution pattern of nutrients in Daya Bay during summer (Han and Ma, 1988; Li et al., 1990; Zhang, 1992; Yang and Tan, 2019). Therefore, the distribution pattern of nutrients in the surface seawater of Daya Bay during summer may be different annually (Shi and Huang, 2013; Wu et al., 2019; Yang and Tan, 2019; Yang et al., 2020; Zhou et al., 2024; Shi et al., 2025; Zhao et al., 2025). Sanmen Island is located in the mouth of Daya Bay and experiences additional influence from the Pearl River plume in summer (Ou et al., 2009; Yang and Tan, 2019; Ma et al., 2025). Compared with historical data, *in situ* environmental parameters showed characteristics of “high temperature, low salinity, low DIN, extremely low ammonia, and extremely low phosphate”, which were distinct from previous data in Daya Bay but frequently in the open sea side of the PRE (Shi and Huang, 2013; Li et al., 2017; Wu et al., 2019; Yang and Tan, 2019; Yang et al., 2020; Tao et al., 2021; Ke et al., 2022; Zhou et al., 2024; Shi et al., 2025; Zhao et al., 2025). Therefore, during the observation, the marine environment around Sanmen Island was likely predominantly influenced by the Pearl River plume, with a relatively weak intrusion of Guangdong coastal upwelling. Furthermore, the unique geographical position of Sanmen Island makes it a potentially critical site for assessing the relative strengths of the Pearl River plume and Guangdong coastal upwelling.

The different response patterns of microorganisms to urea and phosphate addition confirm the serial N limitation pattern in surface seawater near Sanmen Island during summer. Due to the extremely low phosphate concentration during *in situ* observation, the nutrient limitation factor could not be directly calculated from the DIN: DIP ratio. However, chlorophyll *a* concentrations increased in the N group, with a higher increase in the NP group, indicating the nutrient limitation pattern in Sanmen Island was serial N limitation. A similar phenomenon was also observed in the *in situ* observation, where the concentration of chlorophyll *a* increased in Phase 2, coinciding with higher nitrate and nitrite concentrations. In contrast, phosphate addition alone (P group) not only failed to observe increased primary productivity but showed slightly reduced chlorophyll *a* concentration. Since the relative abundance of cyanobacteria did not show a significant correlation with chlorophyll *a* concentrations (*p*-value > 0.05), suggesting the chlorophyll *a* concentration was not only determined by cyanobacteria (**Supplementary Figure 2**). Other phytoplankton also contribute significantly to primary production, such as diatoms. The significant decrease in silicate after the bloom indicates diatoms play an important role in the increase in chlorophyll *a* concentration. Therefore, the rise in chlorophyll *a* concentration may reflect the combined contributions of cyanobacteria, diatoms, and possibly other eukaryotic phytoplankton. However, the increased relative abundance of functional genes derived from cyanobacteria in both N and NP groups confirms that urea addition promoted the growth of primary producers (**Supplementary Table 4**). This provides genetic evidence supporting the serial N limitation pattern observed in surface waters near Sanmen Island during summer.

The bottle effect can alter microbial interactions. The “you produce while I clean up” theory describes an interaction where heterotrophic bacteria (e.g., *Roseobacter*) mineralize organic matter and provide inorganic nutrients, which are then utilized by autotrophs, such as cyanobacteria, for organic matter production (Christie-Oleza et al., 2015). However, in all experimental groups, the addition of exogenous nutrients only enhanced the nutrient uptake potential of microorganisms belonging to one nutritional type of microorganism (autotroph or heterotroph). Even in N and NP groups with higher primary productivity, the nutrient uptake and utilization potential of heterotrophic bacteria were suppressed, likely due to the bottle effect. Since the closed system had almost no material exchange with the outside environment, nutrient supply remained finite over time (Ionescu et al., 2015). As a result, competition for limited resources between autotrophs and heterotrophs inevitably reduced each other’s ecological niches (Calvo-Díaz et al., 2011; Ionescu et al., 2015). Notably, the competitive potential of heterotrophic bacteria was inactivated in the Blank group. Previous studies suggest heterotrophic bacteria exhibit higher phosphorus demand (Biddanda et al., 2001; Makino et al., 2003; Godwin and Cotner, 2015). Phosphate requirements among marine heterotrophic bacteria vary greatly, with their phosphate uptake accounting for 5% to over 90% of total phosphate uptake in the marine environments, depending on the study region (Faust and Correll, 1976; Harrison, 1983; Kirchman, 1994). Overall, heterotrophs dominate phosphate assimilation in aquatic systems (Kirchman, 1994). Therefore, in the P group, the addition of phosphate likely triggered the nutrient competition of heterotrophic bacteria, while cyanobacteria were at a competitive disadvantage due to the lack of available nitrogen sources. In fact, the bottle effect also contributed to differences between the *in situ* environment and the incubation experiment after the increase in primary productivity. During the transition from Phase 1 to Phase 2 in the *in situ* environment, two phenomena occurred that were not observed in the incubation experiment: an increase in DIN concentration and a bloom of *Cyanobium_*PCC-6307-like. These two phenomena were likely caused by the intrusion of an exogenous water mass with higher DIN concentration and another strain of cyanobacteria. This water mass passed through Sanmen Island and mixed with the local water mass. The incubation buckets’ water mass was isolated and therefore unmixed and unaffected.

The variation in relative gene abundances of potassium transporter and potassium channel reflects the importance of potassium ions (K^+^) in microbial survival. K^+^ is involved in many physiological processes, such as microbial osmoregulation, pH maintenance, and enzyme activation (Stautz et al., 2021). Trk/Ktr system is one of the three primary bacterial K^+^ transporter systems (Tanudjaja et al., 2023). In N and NP groups, the relative abundance of cyanobacterial Trk/Ktr genes increased, indicating that the potential demand of cyanobacteria for K^+^ was stimulated by urea addition rather than by the exogenous supply of K^+^. In contrast to Trk/Ktr, the potassium/hydrogen antiporter mediates K^+^ efflux and helps bacteria adapt to alkaline environments (Plack and Rosen, 1980; Radchenko et al., 2006). Given the pH increase in N and NP groups, the increase in relative abundance of potassium/hydrogen antiporter genes derived from cyanobacteria suggests their enhanced potential to alleviate intracellular alkalization stress induced by seawater alkalinization. In the P group, when heterotrophic bacteria (mainly Alphaproteobacteria) gained competitive advantage, the relative abundance of their genes related to K^+^ uptake and efflux increased (**Supplementary Table 4**). Overall, regardless of K^+^ addition, or whether cyanobacteria or heterotrophic bacteria were stimulated, microorganisms with competitive advantage exhibited enhanced potential for both K^+^ uptake and efflux (**Supplementary Table 4**). Considering the important role of K^+^ in maintaining cellular homeostasis, preserving the capacity for K^+^ equilibrium is indispensable for microbial survival (Stautz et al., 2021).

Unclassified Cryomorphaceae and NS4_marine_group had the potential to serve as predictors of eutrophication in the *in situ* environment. Unclassified Cryomorphaceae and NS4_marine_group belong to Flavobacteriales, which have the ability to degrade and utilize algae-derived organic matter, with their abundance showing strong correlations with eutrophication (DeLong et al., 1993; Teeling et al., 2012; Fontanez et al., 2015). In other coastal environments, such as Cam Ranh Bay in Vietnam and the Gulf of Trieste in the northern Mediterranean, NS4_marine_group was identified as a significant predictor of eutrophication or algal blooms, showing strong associations with ammonia and phosphate concentrations (Kolda et al., 2020; Kopprio et al., 2021; Tsang et al., 2021; Celussi et al., 2024). In the first two samples of Phase 2 (L2010 and L2014), the relative abundances of Unclassified Cryomorphaceae and NS4_marine_group increased simultaneously with those of *Cyanobium_*PCC-6307-like, followed by rapid declines. Although object genes derived from Flavobacteriales didn’t dominate in the incubation experiment, the relative gene abundances of *phoD* and *yjbB* increased in the P and NP groups, indicating greater potential for phosphorus acquisition and utilization (Martinez et al., 1996; Clark et al., 1998; Luo et al., 2009; Motomura et al., 2011). Therefore, in the *in situ* environment, Unclassified Cryomorphaceae and NS4_marine_group responded to the bloom of primary productivity, exhibiting a temporary increase in relative abundance.

Cyanobacteria have the potential to promote seawater alkalization through urea decomposition. As dominant primary producers in the ocean, cyanobacteria can utilize urea as a nitrogen source (Esteves-Ferreira et al., 2018; Veaudor et al., 2019; Díez et al., 2023; Li et al., 2023). Urease catalyzes urea hydrolysis to produce ammonia, which could increase environmental pH (Collier et al., 2009; Carlini and Ligabue-Braun, 2016; Veaudor et al., 2019; Li et al., 2023). Urease subunit alpha (*ureC*) is widely used as a representative gene due to its crucial role in urease activity (Reed, 2001; Abdo et al., 2022). In the incubation experiment, both the abundance of *ureC* genes derived from *Synechococcus* and seawater pH increased in N and NP groups (**Supplementary Tables 2**, **4**). Previous studies have confirmed that under acidification conditions, *Synechococcus* strains utilizing urea showed significantly enhanced survival when exogenous urea was provided, whereas *ureC*-mutant strains exhibited reduced tolerance to acidification stress (Li et al., 2023). Although *Synechococcus* were not directly exposed to acidification conditions in the incubation experiment, the increase in *ureC* relative gene abundance and seawater pH in N and NP groups suggests their potential to reduce ocean acidification.

It should be noted that due to the geographical location of Sanmen Island and the variable inter-annual and seasonal dynamics of these transitional waters, the conclusion of Sanmen Island’s nutrient limitation pattern derived from this study, which focused on one site at one season, may not be applicable throughout Daya Bay or across different years and seasons. In addition, due to experimental biases in DNA extraction and sequencing, eukaryotic sequences are exceedingly rare in the sequencing dataset. Therefore, to ensure the reliability of downstream analyses, we excluded eukaryotic sequences. In this study, the absence of eukaryotic sequences limited the exploration of drivers contributing to primary productivity. In future research, the inclusion of eukaryotic sequences will provide a more comprehensive understanding of biogeochemical cycles in transitional water mass.

## 5 Conclusion

In the summer season, as represented by transitional waters off Sanmen Island, the nutrient limitation pattern is characterized by serial nitrogen limitation. The addition of urea enhanced the potential of cyanobacteria to uptake and utilize nutrients, stimulating the bloom in primary productivity. This stimulatory effect was more pronounced under the co-addition of urea and phosphate. In contrast, phosphate addition alone not only failed to increase primary productivity but also increased the competitive potential of heterotrophic bacteria for nutrient acquisition, thereby exacerbating resource competition and constraining the ecological niche of cyanobacteria.

## Data Availability

The datasets presented in this study can be found in online repositories. The names of the repository/repositories and accession number(s) can be found below: https://www.ncbi.nlm.nih.gov/, PRJNA1282918.
