# Supplementary material for: Serial nitrogen-phosphate co-limitation controls the primary productivity in the transitional waters of northern South China Sea and the Pearl River Estuary
Source: Front Microbiomes. 2025 Oct 8;4:1655960. doi: 10.3389/frmbi.2025.1655960 (PMC12993680; doi:10.3389/frmbi.2025.1655960)
Supplement: Supplementary file 1 [file Supplementaryfile1.docx]

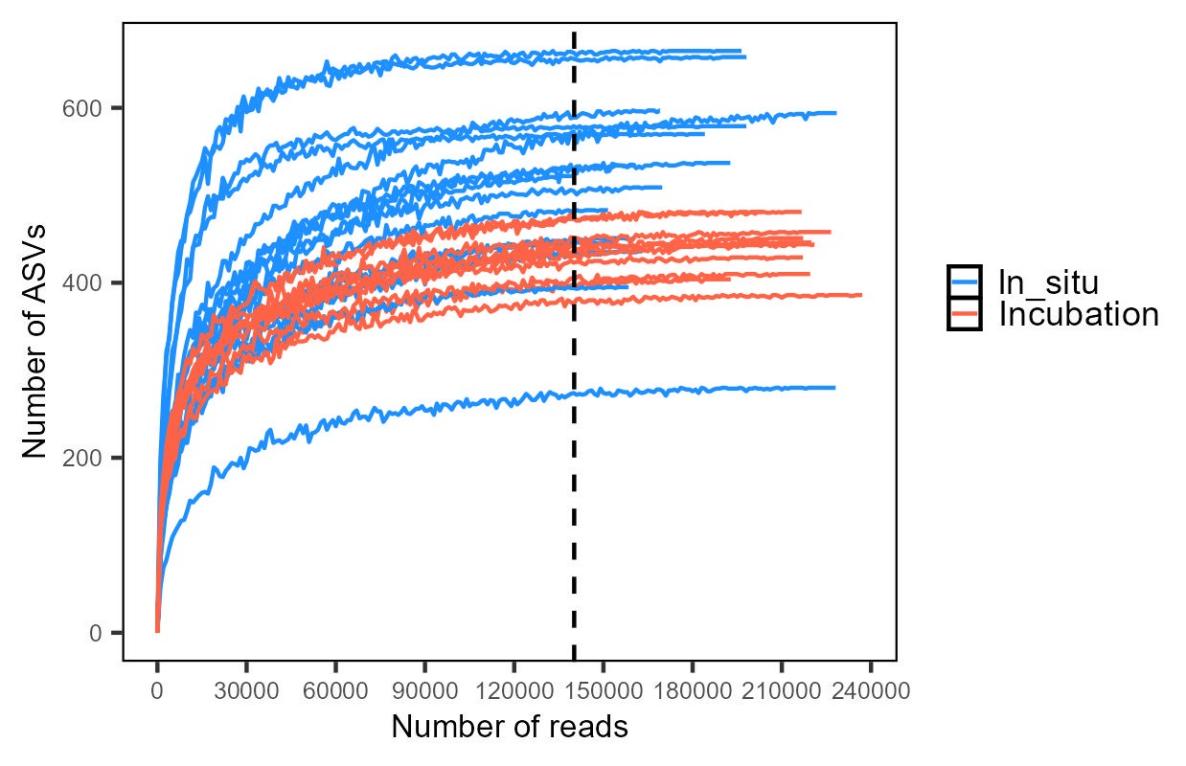


**Supplementary Figure 1** Rarefaction curve. The dashed line in the figure represents the number of reads in the sample with the fewest reads, which is 140,168.


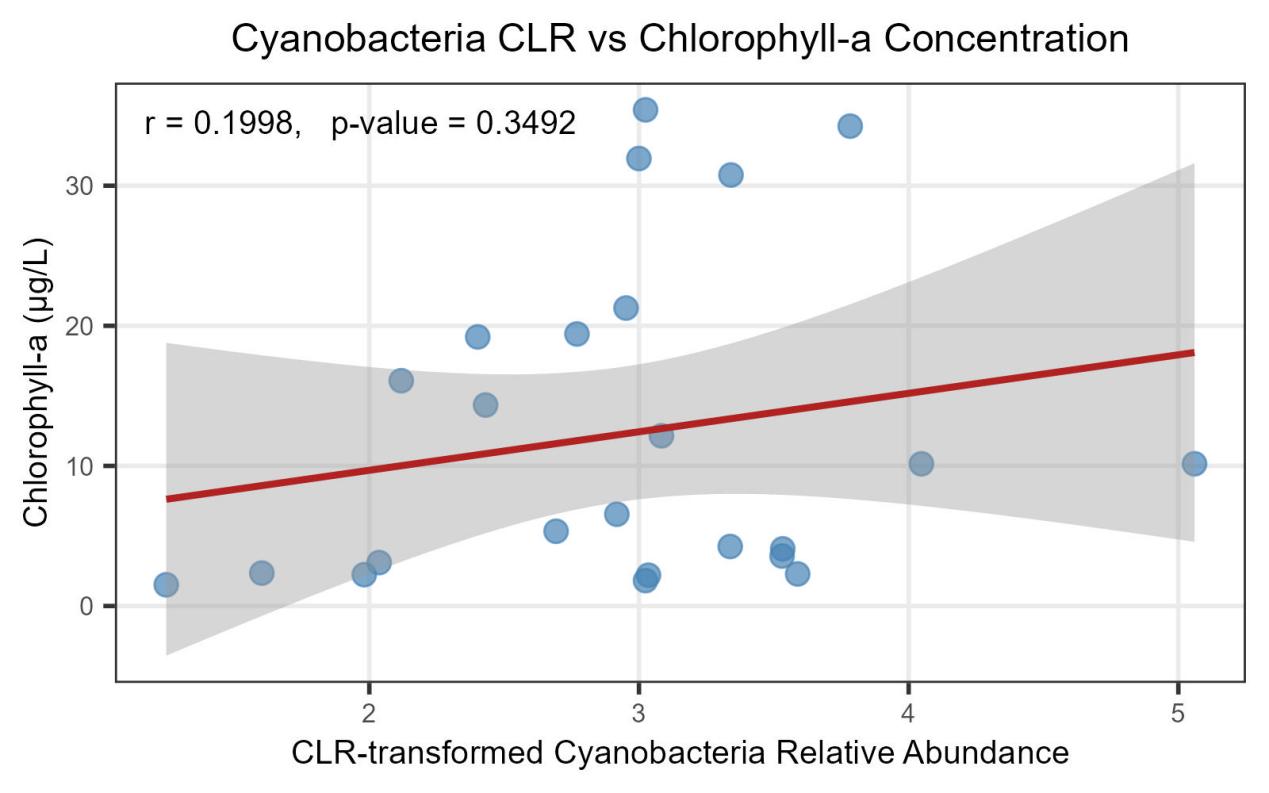


**Supplementary Figure 2** Linear regression between the relative abundance of *Cyanobacteria* (CLR-transformed) and chlorophyll *a* concentration.
